# Melatonin Downregulates PD-L1 Expression and Modulates Tumor Immunity in KRAS-Mutant Non-Small Cell Lung Cancer

**DOI:** 10.3390/ijms22115649

**Published:** 2021-05-26

**Authors:** Yi-Chun Chao, Kang-Yun Lee, Sheng-Ming Wu, Deng-Yu Kuo, Pei-Wei Shueng, Cheng-Wei Lin

**Affiliations:** 1 Department of Biochemistry and Molecular Cell Biology, School of Medicine, College of Medicine, Taipei Medical University, Taipei 110, Taiwan; m120109016@tmu.edu.tw; 2 Graduate Institute of Medical Sciences, College of Medicine, Taipei Medical University, Taipei 110, Taiwan; 3 Shuang Ho Hospital, Taipei Medical University, New Taipei City 235, Taiwan; kangyunlee68@gmail.com (K.-Y.L.); chitosan@tmu.edu.tw (S.-M.W.); 4 Division of Pulmonary Medicine, Department of Internal Medicine, School of Medicine, College of Medicine, Taipei Medical University, Taipei 110, Taiwan; 5 Graduate Institute of Clinical Sciences, College of Medicine, Taipei Medical University, Taipei 110, Taiwan; 6 Division of Radiation Oncology, Far Eastern Memorial Hospital, New Taipei City 220, Taiwan; dykuo@femh.org.tw; 7 Faculty of Medicine, School of Medicine, National Yang Ming Chiao Tung University, Taipei 112, Taiwan; 8 Cell Physiology and Molecular Image Research Center, Wan Fang Hospital, Taipei Medical University, Taipei 110, Taiwan; 9 Drug Development and Value Creation Research Center, Kaohsiung Medical University, Kaohsiung 817, Taiwan

**Keywords:** melatonin, lung cancer, PD-L1, tumor immunity, Hippo pathway

## Abstract

Non-small cell lung cancer (NSCLC) patients harboring a KRAS mutation have unfavorable therapeutic outcomes with chemotherapies, and the mutation also renders tolerance to immunotherapies. There is an unmet need for a new strategy for overcoming immunosuppression in KRAS-mutant NSCLC. The recently discovered role of melatonin demonstrates a wide spectrum of anticancer impacts; however, the effect of melatonin on modulating tumor immunity is largely unknown. In the present study, melatonin treatment significantly reduced cell viability accompanied by inducing cell apoptosis in KRAS-mutant NSCLC cell lines including A549, H460, and LLC1 cells. Mechanistically, we found that lung cancer cells harboring the KRAS mutation exhibited a higher level of programmed death ligand 1 (PD-L1). However, treatment with melatonin substantially downregulated PD-L1 expressions in both the presence and absence of interferon (IFN)-γ stimulation. Moreover, KRAS-mutant lung cancer cells exhibited higher Yes-associated protein (YAP) and transcriptional coactivator with PDZ-binding motif (TAZ) levels, and PD-L1 expression was positively correlated with YAP and TAZ in lung cancer cells. Treatment with melatonin effectively suppressed YAP and TAZ, which was accompanied by downregulation of YAP/TAZ downstream gene expressions. The combination of melatonin and an inhibitor of YAP/TAZ robustly decreased YAP and PD-L1 expressions. Clinical analysis using public databases revealed that PD-L1 expression was positively correlated with YAP and TAZ in patients with lung cancer, and PD-L1 overexpression suggested poor survival probability. An animal study further revealed that administration of melatonin significantly inhibited tumor growth and modulated tumor immunity in a syngeneic mouse model. Together, our data revealed a novel antitumor mechanism of melatonin in modulating the immunosuppressive tumor microenvironment by suppressing the YAP/PD-L1 axis and suggest the therapeutic potential of melatonin for treating NSCLC.

## 1. Introduction

Lung cancer is still the leading cause of cancer-related mortality, and non-small cell lung cancer (NSCLC) accounts for 85% of all lung cancers [1]. Oncogenic KRAS mutations are a major driver of NSCLC, accounting for over 30% of NSCLC cases in Western countries and 10% of total lung cancer cases in Asia [2]. NSCLC patients with a KRAS mutation show a poor response to front-line chemotherapy, and they often exhibit worse prognoses. Disappointingly, unlike other oncoproteins, the mutant KRAS protein is thought to be an undruggable target due to various conformations of its mutant types [2,3]. Recently, immune checkpoint blockage through targeting programmed death-ligand 1 (PD-L1) and by its receptor programmed death-1 (PD-1), has changed the treatment paradigm for lung cancer patients, especially for those harboring a KRAS mutation [4]. KRAS mutations are often associated with high tumor mutation burdens, elevated PD-L1 expression, and an immunosuppressive tumor microenvironment [5,6]. Mutations of KRAS facilitate immune evasion by orchestrating a pro-inflammatory microenvironment [7], and evidence indicates that oncogenic RAS promotes stabilization and upregulation of PD-L1 [8]. Notably, several studies showed that patients with KRAS mutations are more sensitive to PD1/PD-L1 inhibitors [9], suggesting that the KRAS mutation may be vulnerable to immunotherapy.

Melatonin (N-acetyl-5-methoxytryptamine), an indolamine that is primarily synthesized and secreted by the pineal gland, has various biological functions such as regulation of circadian rhythm, as well as antioxidant, anti-aging, and antitumor activities [10]. There is a large amount of evidence on the benefits of melatonin in anticancer properties including its pro-apoptotic, anti-proliferative, anti-angiogenic, and anti-metastatic properties [11,12,13]. Melatonin also has a synergistic effect with different chemotherapeutic drugs [14,15,16]. In the clinical study, NSCLC patients concomitantly treated with melatonin exhibited better survival outcomes compared to those treated with chemotherapy alone [17]. Moreover, melatonin showed an anti-inflammatory response, which is known to be essential for promoting tumor development [18]. Melatonin also showed immunomodulatory activity in solid tumors [19]. Despite the antitumor activities of melatonin having been widely reported, the effect of melatonin on tumor immunity and the mechanism involved in the process remain unclear. 

Yes-associated protein (YAP) and transcriptional coactivator with the PDZ-binding motif (TAZ) are transducers of the Hippo pathway, which controls organ development and whose dysregulation is involved in tumorigenesis [20,21]. YAP and TAZ are often upregulated or hyperactivated in many solid tumors including NSCLC [22,23]. Elevation of YAP/TAZ promotes stemness of NSCLC and contributes to the resistance of NSCLC to chemo- and target therapies [24,25]. On the other hand, inhibition of YAP/TAZ increases cytotoxicity of chemotherapy and sensitivity to various target therapies including EGFR-TKIs and MEK inhibitors in NSCLC [26,27]. YAP/TAZ also participates in KRAS oncogene-driven pancreatic ductal carcinoma [28], and KRAS and YAP signaling pathways converge to regulate the epithelia-mesenchymal transition in lung cancer [29]. These data indicate that activation of YAP/TAZ plays a crucial role in KRAS oncogene-mediated tumorigenesis. However, no study has reported on the role of melatonin in Hippo-YAP signaling for treatment of KRAS-mutant lung cancer.

Herein, we investigated the inhibitory mechanism of melatonin against NSCLC and tumor immunity. We show for the first time that melatonin attenuates PD-L1 expression through suppressing YAP/TAZ and thereby modulating tumor immunity in KRAS-mutant NSCLC. We demonstrate that melatonin modulates an immunosuppressive tumor microenvironment, and our findings suggest a better understanding of the antitumor activity of melatonin in the surrounding tumor microenvironment.

## 2. Results

### 2.1. Melatonin Reduces the Viability and Induces Apoptosis in KRAS-Mutant NSCLC Cells

**Figure 1:**
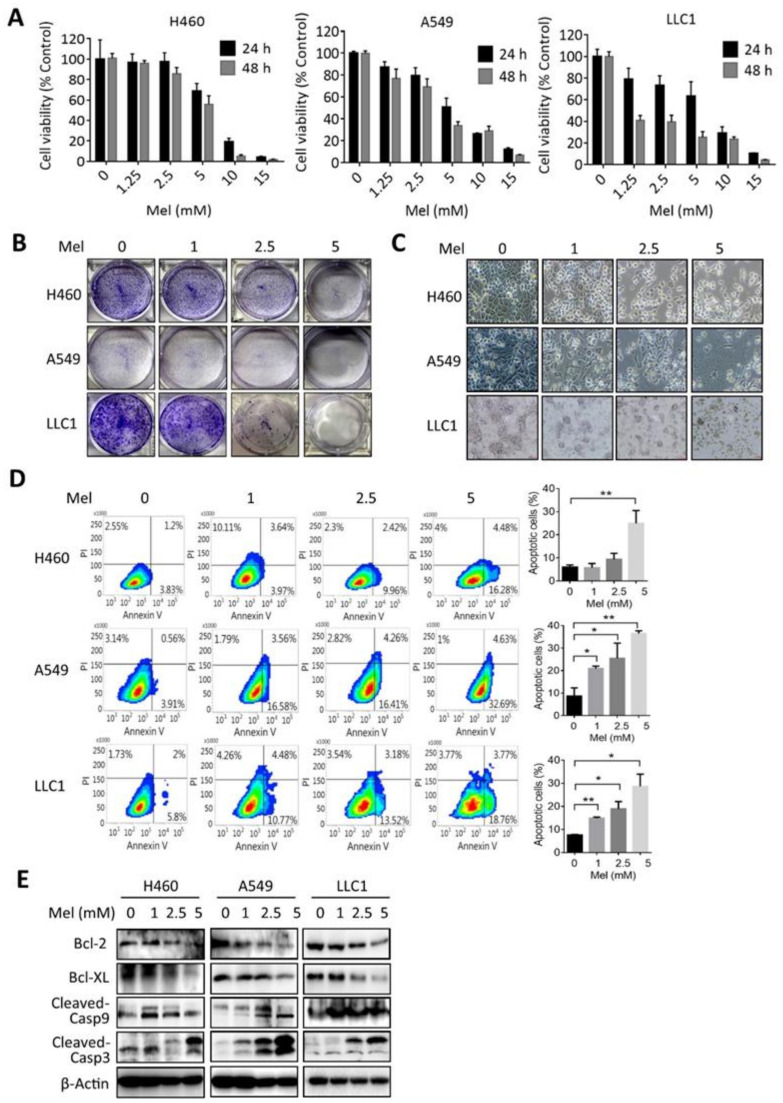
Effect of melatonin on the viability of NSCLC. (**A**) H460, A549, and LLC1 cells were treated with various concentrations of melatonin (Mel: 0, 1.25, 2.5, 5, 10, 15 mM) for 24 and 48 h, and cell viability was measured with a CCK8 assay. Data are expressed as a percentage of the control. Values are expressed as the mean ± standard deviation. (**B**) Colony formation of H460, A549, and LLC1 cells in the presence of melatonin (Mel: 0, 1, 2.5, 5 mM) for 7 days. (**C**) Microscopic observation of cell morphologies of H460, A549, and LLC1 cells in the presence of melatonin (Mel: 0, 1, 2.5, 5 mM) for 24 h. (**D**) Flow cytometry analysis of Annexin V/PI staining in response to melatonin (Mel: 0, 1, 2.5, 5 mM) for 24 h. Quantifications of the total apoptotic cell population (Annexin V + /PI- and Annexin V + /PI + ) were obtained from three independent experiments (right panel). Data are presented as the mean ± standard deviation (SD).* *p* < 0.05, ** *p* < 0.01, as determined by an unpaired *t*-test. (**E**) Western blot analysis of apoptosis-related proteins in H460, A549, and LLC1 cells in response to melatonin (Mel: 0, 1, 2.5, 5 mM) for 24 or 48 h.

To examine the effects of melatonin on lung cancer, human NSCLC cell lines including the H460 and A549, and mouse Lewis cell carcinoma LLC1 cell lines, which harbor KRAS mutations, were incubated with different concentrations of melatonin for 24 and 48 h. Results of cell viability and colony formation assays showed that melatonin dose-dependently reduced viability of lung cancer cells (Figure 1A,B). Microscopic observation showed that treatment with melatonin at 1 mM induced cellular shrinkage, while promoting the formation of an apoptotic-like morphology under 2.5 and 5 mM of melatonin treatment (Figure 1C). Results of flow cytometry analysis further confirmed that treatment with melatonin at 2.5 and 5 mM concentrations increased Annexin V-positive cells (Figure 1D), and Western blot analysis showed that incubation with melatonin decreased anti-apoptotic proteins levels, including Bcl2 and Bcl-xL, but increased cleavages of Caspase 9 and Caspase 3 (Figure 1E). These data suggest that melatonin induces the death of NSCLC cells.

### 2.2. Melatonin Downregulates PD-L1 Expression in KRAS-Mutant NSCLC Cells

**Figure 2:**
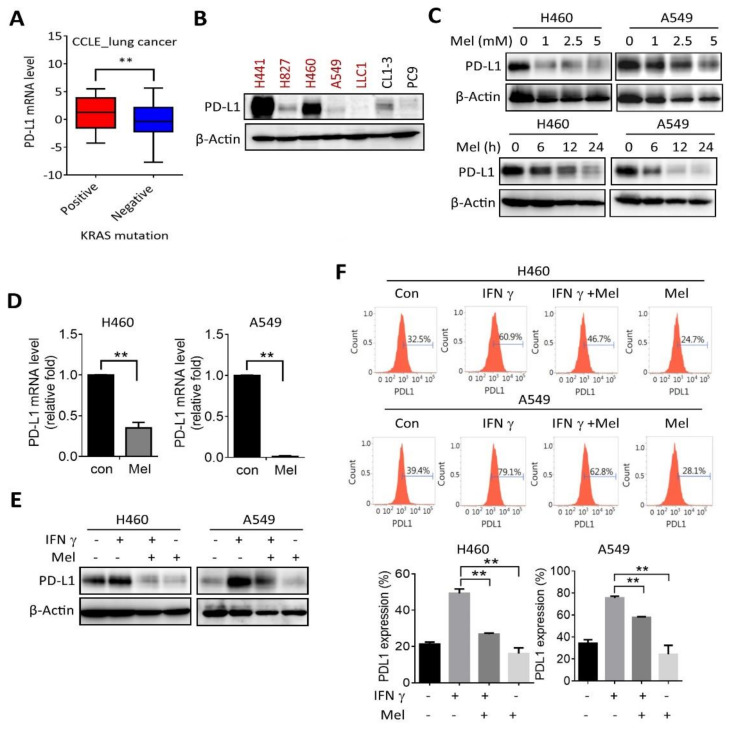
Melatonin downregulates PD-L1 in NSCLC. (**A**) Box plot with Tukey whisker of PD-L1 mRNA level in KRAS mutant and wild-type lung cancer cells. ** *p* < 0.01 as determined by an unpaired *t*-test. (B) Western blot analysis of PD-L1 protein level in NSCLC cell lines. KRAS-mutant cell lines were labeled in red color. (**C**) H460 and A549 cells were treated with various concentrations of melatonin (Mel: 0, 1, 2.5, 5 mM) for 24 h (upper panel), or treated with 2.5 mM melatonin at different time intervals (lower panel), and protein lysates were subjected to Western blot analysis. (**D**) Real-time PCR analysis of PD-L1 mRNA expression in response to melatonin (Mel: 2.5 mM) for 6 and 12 h in A549 and H460 cells, respectively. Values are expressed as the mean ± standard deviation. ** *p* < 0.01 was regarded as indicating a significant difference. (**E** and **F**) Serum-starved H460 and A549 cells were pretreated with melatonin (2.5 mM) for 60 min followed by stimulation with IFN γ (50 ng/mL) for a further 24 h. (**E**) Protein level of PD-L1 in total cell lysate was measured by Western blot, and (**F**) histogram plot of PD-L1 expression was performed by flow cytometry analyses. Quantifications of PD-L1 positive percentages from three-independent experiments are shown (lower panel). ** *p* < 0.01, as determined by an unpaired *t*-test.

KRAS mutations are associated with an immunosuppressive tumor microenvironment. To evaluate the role of melatonin in modulating tumor immunity, we analyzed the level of the immune checkpoint molecule PD-L1 in lung cancer cells using the Cancer Cell Line Encyclopedia (CCLE) database. Results showed that the mRNA expression of PD-L1 (*CD274*) was higher in KRAS-mutant lung cancer cells than in KRAS wide-type (WT) cells (Figure 2A), and Western blot analysis showed a higher tendency of PD-L1 protein level in KRAS mutant H441 and H460 cells (Figure 2B). Importantly, treatment with melatonin dose- and time-dependently suppressed PD-L1 protein levels in H460 and A549 cells (Figure 2C). Melatonin treatment substantially decreased the PD-L1 protein level within 24 h (Figure 2C). Additionally, the mRNA level of PD-L1 was downregulated after incubation with melatonin for 6–12 h (Figure 2D). Moreover, pretreatment with melatonin impeded IFNγ-induced PD-L1 upregulation, as validated by Western blot and flow cytometry analyses (Figure 2E,F).

### 2.3. Melatonin Downregulates the YAP/PD-L1 Axis

**Figure 3:**
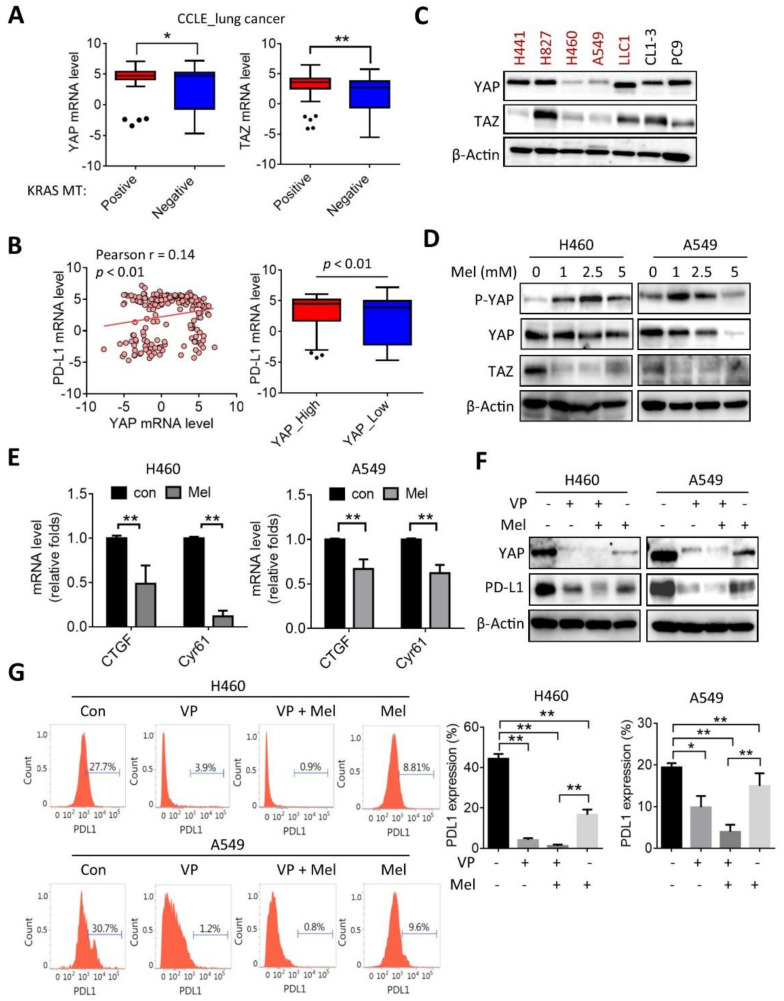

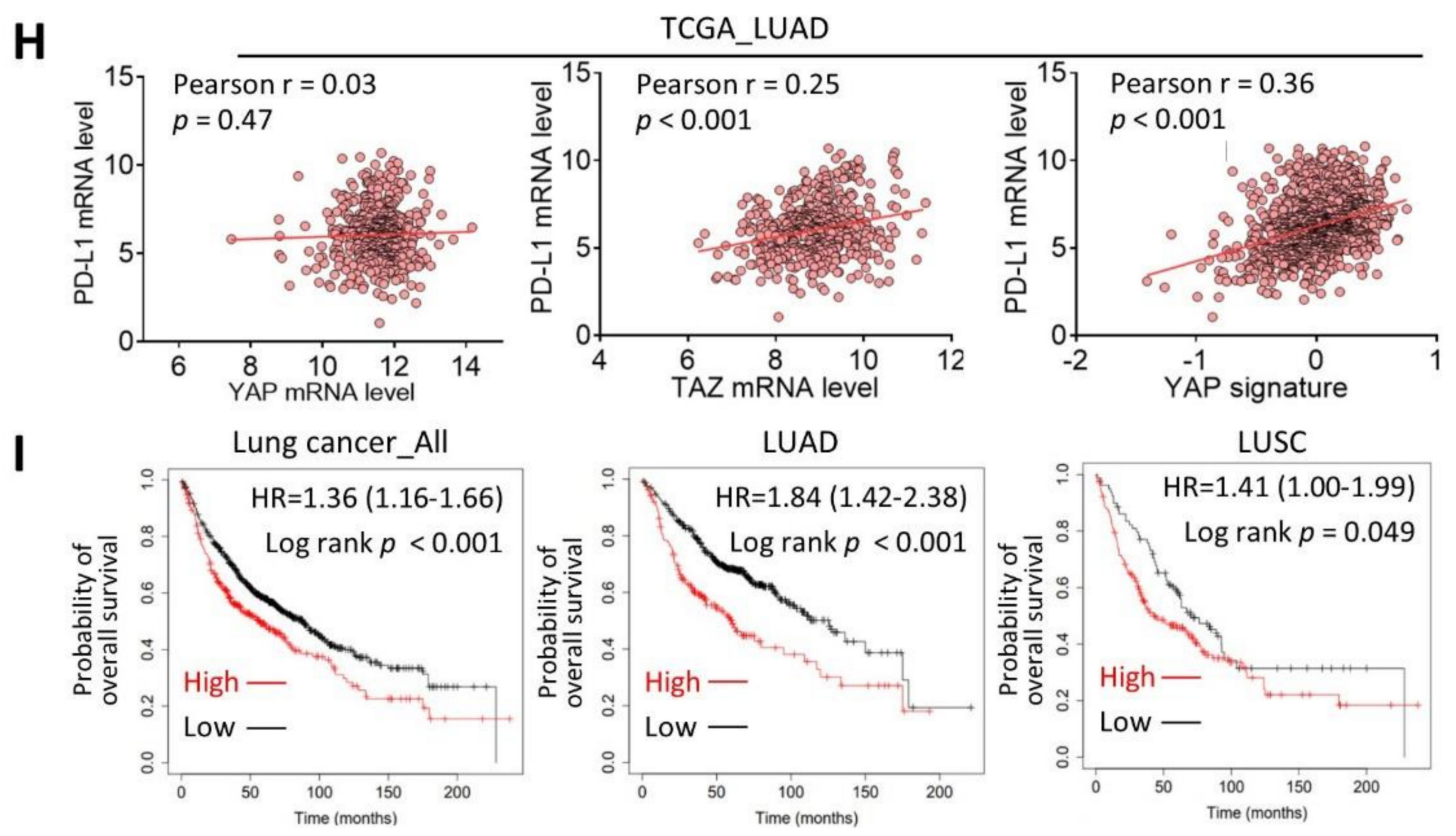
Melatonin inhibits the YAP/PD-L1 axis. (**A**) Box plots with Tukey whisker of YAP (*YAP1*) and TAZ (*WWTR1*) mRNA levels in KRAS-mutant and wild-type lung cancer cell lines. * *p* < 0.05 and ** *p* < 0.01 as determined by an unpaired *t*-test. (**B**) Scatterplot of PD-L1 (*CD274*) and YAP (*YAP1*) in lung cancer cell lines (left panel). Correlation coefficient was performed by Pearson’s test. Box blot of PD-L1 (*CD274*) levels in lung cancer cell lines stratified by YAP (*YAP1*) expression levels (right panel). (**C**) Western blot analysis of YAP and TAZ protein levels in NSCLC cell lines. KRAS-mutant cell lines were labeled in red color. (**D**) H460 and A549 cells were treated with various concentrations of melatonin (Mel: 0, 1, 2.5, 5 mM) for 24 h, and protein lysates were subjected to Western blot analysis. (**E**) RT-qPCR analysis of the expressions of *CTGF* and *Cyr61* in melatonin-treated H460 and A549 cells. ** *p* < 0.01 as determined by an unpaired *t*-test. (**F**) Western blot analysis of YAP and PD-L1 protein levels in H460 and A549 cells treated with verteporfin (VP: 1 μM) and melatonin (Mel: 2.5 mM). (**G**) Flow cytometry analysis of PD-L1 expression in H460 and A549 treated with verteprofin (VP: 1 µM) and melatonin (Mel: 2.5 mM). Quantifications of PD-L1 positive percentage from three-independent experiments are shown. * *p* < 0.05 and ** *p* < 0.01, as determined by a one-way ANOVA. (**H**) Scatterplot of PD-L1 (*CD274*) and YAP (*YAP1*), TAZ (*WWTR1*), and YAP/TAZ signature in TCGA_LUAD cohort. Correlation coefficients were determined by Pearson’s test. (**I**) Kaplan-Meier curve analysis of the overall survival probability of lung cancer patients stratified by PD-L1 (*CD274*) expression level. LUAD: lung adenocarcinoma; LUSC: lung squamous cell carcinoma. HR: hazard ratio.

To explore the inhibitory mechanism by which melatonin attenuates PD-L1 expression further, we analyzed YAP and TAZ expressions in lung cancer cells. YAP plays a crucial role in KRAS-mutation-driven tumorigenesis of pancreatic ductal adenocarcinoma [30], and we previously identified that YAP/TAZ expressions participate in the resistance of anti-EGFR therapy in KRAS-mutant NSCLC [26]. Analysis of mRNA levels from the CCLE database showed that the expressions of YAP (*YAP1*) and TAZ (*WWTR1*) were upregulated in KRAS-mutant lung cancer cells, compared to KRAS wild-type tumor cells (Figure 3A). Moreover, expression of PD-L1 positively correlated with YAP/TAZ in lung cancer cells (Figure 3B). We further analyzed protein levels of YAP/TAZ in lung cancer cell lines, and the results showed that YAP and TAZ were not exclusively expressed in KRAS-mutant lung cancer cells (Figure 3C), suggesting that other molecules apart from KRAS may regulate protein levels of YAP/TAZ. Nevertheless, we found that treatment with melatonin at 1–2.5 mM concentrations for 24 h induced phosphorylation of YAP and was accompanied with a reduction in total YAP protein level by 5 mM melatonin treatment in both H460 and A549 cells (Figure 3D). The phosphorylated YAP induces cytoplasmic retention and subsequently facilitates proteasomal degradation. In addition, we also found that treatment with melatonin substantially inhibited TAZ protein levels in H460 and A549 cells (Figure 3D). Results of real-time PCR assays also showed that melatonin downregulated mRNA levels of YAP/TAZ downstream targets including *CTGF* and *Cyr61* (Figure 3E). Additionally, we validated that treatment with the inhibitor of YAP/TAZ by verteporfin (VP) caused robust decreases in YAP and PD-L1 protein levels, and co-treatment with VP and melatonin enhanced the inhibitory effect, as measured by Western blot and flow cytometry analyses (Figure 3F,G). Furthermore, examination of a TCGA lung adenocarcinoma cohort validated that PD-L1 (*CD274*) was positively correlated with YAP (*YAP1*), TAZ (*WWTR1*), and the YAP/TAZ signature (Figure 3H), and NSCLC patients with high PD-L1 expression conferred poor survival probabilities (Figure 3I). These data suggest that elevation of YAP/TAZ in KRAS-mutant NSCLC plays a role in PD-L1 regulation, and melatonin effectively suppressed the YAP/PD-L1 axis in KRAS-mutant NSCLC cells. 

### 2.4. Melatonin Suppresses Tumor Growth and Exerts Antitumor Immunity

**Figure 4:**
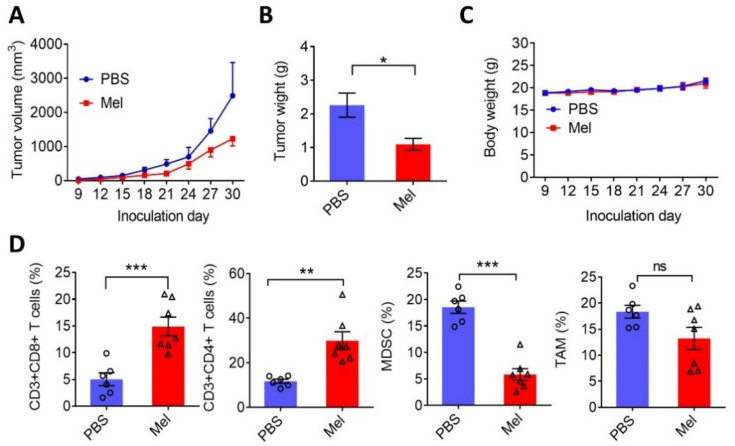
Effects of melatonin on tumor growth and tumor immunity. (**A**) Growth curve of LLC1 tumor-bearing mice administrated PBS (*n* = 6) or melatonin (30 mg/kg, three times per week, *n* = 7) for 4 weeks. Tumor volumes were measured every other day. (**B**) Tumor weights were measured at the end of the experiment. (**C**) Mouse body weight changes under different treatments. (**D**) Quantification of tumor-infiltrating lymphocytes in PBS- and melatonin-treated tumor tissues, as measured by flow cytometry analysis. Values are expressed as the mean ± standard deviation. * *p* < 0.05, ** *p* < 0.01, *** *p* < 0.001 indicating a significant difference.

We next evaluated the effect of melatonin on tumor immunity using a syngeneic mouse model. B57CL/6 mice were subcutaneously inoculated with LLC1 tumor cells and administered melatonin (30 mg/kg, three times per week) for a total of four weeks. Results showed that mice receiving melatonin exhibited effective inhibition of tumor growth (Figure 4A,B), whereas the mice body weights did not significantly change during melatonin administration (Figure 4C). Notably, analyses of tumor-infiltrating lymphocytes indicated that treatment with melatonin significantly increased the number of CD3 + CD4+ and CD3 + CD8+ T cells, but reduced infiltration of Ly6G + F4/80- myeloid-derived suppressor cells (MDSC). Melatonin treatment slightly but insignificantly reduced F4/80+ tumor-associated macrophages (TAM) (Figure 4D and Figure S1). These data indicate that melatonin may suppress tumor growth and ameliorate the immunosuppressive tumor microenvironment. 

## 3. Discussion

Immunotherapies targeting PD-L1 or its receptor PD1, have shown benefits in patients with advanced lung cancer. Evidence indicates that oncogenic RAS promotes stabilization of PD-L1, and KRAS mutations upregulate PD-L1 and mediate immune escape in NSCLC [8,31]. Although KRAS mutations facilitate immune evasion by promoting a pro-inflammatory microenvironment, several clinical studies also reported that patients with KRAS mutations were more sensitive to PD1/PD-L1 inhibitors, suggesting vulnerability to anti-PD1/PD-L1 therapy by patients with KRAS mutations. In our study, the PD-L1 mRNA expression was associated with mutant KRAS by the CCLE database; however, protein levels of PD-L1 were not exclusively expressed in mutant KRAS cells. Several studies and meta-analyses have confirmed the correlation between PD-L1 expression and *KRAS* status in NSCLC [32,33,34,35], but conflicting studies reported that expression of PD-L1 was varied according to the pattern of downstream signaling [36,37]. Moreover, other oncogenic drivers such as *BRAF*, *PIK3CA*, and *MET* showed more frequent PD-L1 expression [38]. Therefore, it is warranted to clarify the regulation of PD-L1 expression in NSCLC.

Possible crosstalk between melatonin and the Hippo-YAP signaling pathway involves the G protein-coupled receptor (GPCR), which is mainly associated with the melatonin receptor and is also involved in the regulation of the upstream regulators of YAP/TAZ, such as LATS1/2 and F-actin [39]. Previous studies reported that melatonin protects against lung fibrosis by inhibiting the Hippo-YAP pathway [40]. The effect of melatonin on the YAP/TAZ pathway in lung cancer remains unclear. In the present study, we identified that YAP/TAZ expressions were upregulated in KRAS-mutant lung cancer cells. Moreover, we demonstrated that melatonin effectively suppressed YAP/TAZ in KRAS-mutant A549 and H460 cells, and melatonin downregulated the YAP/TAZ downstream genes’ expression. Additionally, we found that melatonin induced phosphorylation of YAP. Phosphorylation of YAP and TAZ by LATS1/2 results in their cytoplasmic retention and facilitates proteasomal degradation. Because YAP/TAZ also play crucial role in promoting tumor growth, the inhibition of YAP/TAZ results in inducing cell death [25,26,41]. Therefore, downregulation of YAP/TAZ may contribute to melatonin-induced growth inhibition. Nevertheless, it is still unclear whether melatonin affects upstream molecules of the Hippo cascade which is worth exploring.

Activation of YAP/TAZ has been reported to regulate PD-L1 transcription and immune escape [42], while suppression of the Hippo-YAP signaling attenuates PD-L1 expression and shows promise in ameliorating immune evasion [43]. It has been reported that melatonin can stimulate nature killer (NK) cells and modulate regulatory T (Treg) cells, and melatonin promotes Th1 in tumor-bearing mice [44,45,46]. A previous study reported that incubation of macrophages with exosome from melatonin-treated hepatocarcinoma cells downregulated PD-L1 and proinflammatory cytokines’ expressions [47]. The combined use of melatonin and an indoleamine 2,3-dioxygenase-1 inhibitor improved the efficacy of immunotherapy (gDE7) targeting human papillomavirus (HPV)-associated tumors [48]. Moreover, melatonin suppresses pro-inflammatory responses; thus, melatonin also plays a crucial role in promoting the development of an immunosuppressive tumor microenvironment [49]. However, the effect of melatonin against tumor PD-L1 expression and the underlying mechanism remain unclear. In the present study, we demonstrated that melatonin suppressed YAP/TAZ which was accompanied by inhibition of PD-L1. Concomitant treatment with melatonin and a YAP/TAZ inhibitor synergistically suppressed PD-L1 expression in NSCLC cells. The in vivo study revealed that melatonin suppressed the expressions of YAP and PD-L1 in tumor tissues, and administration of melatonin decreased infiltrations of TAM and MDSC while increasing CD8+ and CD4+ T cells. Because melatonin shows a broad synergistic benefit with chemotherapies, the therapeutic efficacy of melatonin as an adjuvant, in combination with immunotherapies such as anti-PD1 or anti-PD-L1, envisions a promising approach for lung cancer treatment, and future research is warranted to investigate.

## 4. Materials and Methods

### 4.1. Cell Culture

The human A549, H460, PC9, H827, H441, and mouse Lewis cell carcinoma LLC1 NSCLC cell lines were purchased from the Bioresource Collection Research Center (BCRC, Hsinchu, Taiwan) or American Type Culture Collection (ATCC, Manassas, VA, USA). Genetic identities of the cell lines were authenticated through short tandem repeat profiling (GenePrint 10 System). Cells were maintained in the Roswell Park Memorial Institute (RPMI) 1640 medium supplemented with 7% fetal bovine serum (FBS), 1% penicillin-streptomycin, and 1% Glutagro (Corning, Corning, NY, USA). All cells were cultured at 37 °C and in a humidified atmosphere of 5% CO_2_.

### 4.2. Cell Viability Assay

NSCLC cells (6 × 10^3^ cells/well) were seeded into 96-well plates and treated with different concentrations of melatonin (Selleck) for 24 and 48 h. After incubation, cells were fed growth medium containing the Cell Counting Kit-8 (CCK-8) reagent (Dojindo Molecular Technologies) for 60 min, and the formazan dye was measured with a spectrophotometer at 450 nm absorbance. Cell viability is expressed as the percentage of untreated control cells.

### 4.3. Flow Cytometry

To detect cell apoptosis, NSCLC cells (5 × 10^5^ cells/well) were seeded into 6-well plates and treated with melatonin for 24 h. Cells were trypsinized followed by incubation with an Annexin V-FITC/propidium iodide (PI) Apoptosis detection kit (Elabscience) according to the manufacturer’s instruction. To measure PD-L1 protein levels, cells were treated with melatonin (2.5 mM) in the presence or absence of IFNγ (50 ng/mL) for 24 h. Cells were then trypsinized followed by incubation with a PE-conjugated anti-PD-L1 antibody (Abcam) and analyzed with a BD FACSVia flow cytometer (BD Biosciences).

### 4.4. Western Blotting

Cells were lysed in an ice-cold radioimmunoprecipitation assay (RIPA) buffer supplemented with a protease and phosphatase inhibitor cocktail (Roche, Mannheim, German). Equal amounts of protein were separated by sodium dodecylsulfate (SDS)-polyacrylamide gel electrophoresis (PAGE) and transferred to polyvinylidene difluoride membranes (Millipore, Bedford, MA, USA). Membranes were blocked with 1% bovine serum albumin/TBST blocking buffer at room temperature for 30 min and then incubated overnight at 4 °C with specific primary antibodies. Primary antibodies for YAP (#12395), YAP/TAZ (#8410), phospho-YAP(S127) (#13008), Caspase 9 (#9504), and PD-L1 (#13684) were purchased from Cell Signaling Technology (Danvers, MA, USA). Antibodies for β-actin (GTX109639), Bcl-2 (GTX100064), Bcl-xL (GTX105661), Caspase 3 (GTX110543), and Caspase 9 (GTX112888) were obtained from GeneTex (San Antonio, TX, USA). Membranes were washed three times with the TBST wash buffer followed by incubation with a horseradish peroxidase-conjugated secondary antibody (Jackson ImmunoResearch, West Grove, PA, USA) at room temperature for 1 h. Bands were detected with an enhanced chemiluminescence (ECL) system (Millipore, Bedford, MA, USA). Western blotting was performed at least three times, and representative experiments are shown.

### 4.5. RNA Isolation and Real-Time Quantitative Polymerase Chain Reaction

Total RNA was extracted with a GENzolTM TriRNA Pure kit (Geneaid, Taipei, Taiwan). cDNA was synthesized with M-MLV reverse transcriptase (Promega) and amplified with the GoTaq qPCR Master Mix (Promega) in a StepOne Plus Real-Time PCR system (Applied Biosystems, Darmstadt, Germany) with specific primers as follows: *Cyr61*, 5′-GAGTGGGTC TGTGAC GAGGAT-3′(sense), and 5′-GGTTGTATAGGATGCGAGGCT-3′(antisense); *CTGF*, 5′-CTGTGCAGCATGGACGTT-3′ (sense), and 5′-GGCAGC TTGACCCTCCTC-3′ (antisense); *PD-L1*, 5′-GTGGCATCCAAGATACAAACTCAA-3′ (sense), and 5′-TCCTTCCTCTTGTCACGCTCA-3′ (antisense). Results were calculated using the ΔΔCT equation and are expressed as multiples of change relative to a control sample [50].

### 4.6. Animal Study

All animals were cared for in a specific pathogen-free room and treated following the animal care protocol approved by an animal committee. Mouse LLC1 (1 × 10^6^) cells were subcutaneously injected into 8-week-old -C57BL/6 mice. After one week of inoculation, the mice were intraperitoneally administered melatonin (30 mg/kg, three times per week) for 4 weeks. The tumor volume and mice body weights were monitored every 2 days during the experiments. Tumor volume was calculated using the following equation: length × (width)2 × 0.5. To analyze tumor-infiltrating lymphocytes (TILs), freshly isolated tumor tissue was cut into small pieces and disassociated by the gentleMACS tumor dissociation kit (Miltenyi Biotec). The suspension was further treated with a RBC lysis buffer to remove red blood cells. Approximate 1 × 10^6^ isolated cells were incubated with fluorophore-conjugated antibodies including CD45-APC, CD11b-PE, CD3-FITC, CD8-APC-Vio770, Ly6G-FITC, CD4-PE-Vio770, and F4/80-APC (Miltenyi Biotec) and analyzed by the BD FACSVia flow cytometer (BD Biosciences) [43].

### 4.7. Statistical Analyses

Gene expression patterns of PD-L1 (*CD274*), YAP (*YAP1*), and TAZ (*WWTR1*) were downloaded from the University of California, Santa Cruz (UCSC) Xena browser (https://xenabrowser.net/ accessed on 5 May 2021) and Cancer Cell Line Encyclopedia (CCLE) (https://portals.broadinstitute.org/ccle accessed on 5 May 2021). The unit of mRNA in CCLE is log2 normalized RNA expression, and the unit of gene expression in the TCGA dataset is (norm_count + 1). The YAP signature was defined by using the CORDENONSI_YAP_CONSERVED signature from a gene set enrichment analysis (GSEA). Survival prognoses of PD-L1 (*CD274*) in lung cancer patients were evaluated using the KM plotter (https://kmplot.com accessed on 5 May 2021). Data are presented as the mean ± standard deviation (SD) of three independent experiments. Statistical significance was determined by an unpaired, two-tailed Student’s *t*-test unless stated otherwise. * *p* < 0.05 and ** *p* < 0.01 indicate statistical significance. Correlation coefficients were analyzed by Pearson’s test. All statistical analyses were carried out using GraphPad Prism 6.0 software.

## 5. Conclusions

Taken together, our findings provide a novel understanding of the antitumor activity of melatonin by suppressing the YAP/PD-L1 axis and enhancing antitumor immunity. Because melatonin shows broad synergistic benefits with chemotherapies, the therapeutic potentiality of melatonin as an adjuvant in combination with immunotherapy envisions a promising approach for cancer treatment.

## Data Availability

Not applicable.

## Supplementary Materials

The following are available online at https://www.mdpi.com/article/10.3390/ijms22115649/s1, Figure S1: Gating Strategy for tumor-infiltrating lymphocytes.
