# Supplementary figures and images for: Melatonin Downregulates PD-L1 Expression and Modulates Tumor Immunity in KRAS-Mutant Non-Small Cell Lung Cancer
Source: Int J Mol Sci. 2021 May 26;22(11):5649. doi: 10.3390/ijms22115649 (PMC8199131; doi:10.3390/ijms22115649)

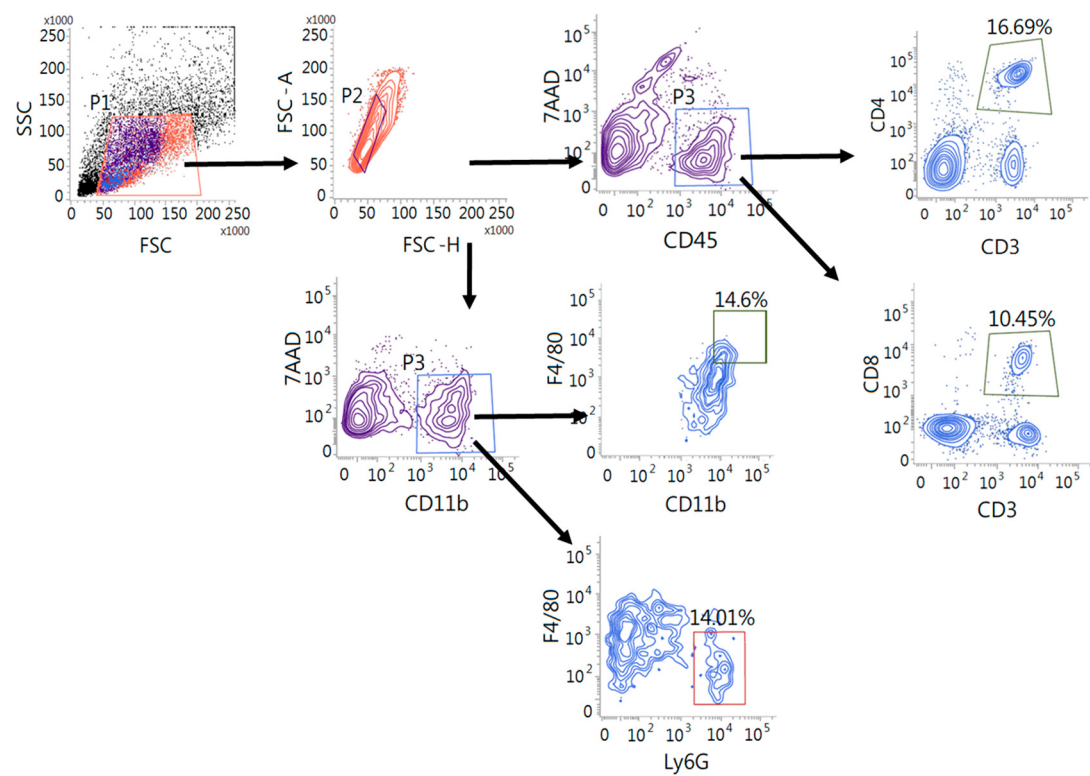

Supplemental figure 1. Gating Strategy for tumor-infiltrating lymphocytes

Supplement: Supplementary file 1 [file ijms-22-05649-s001.zip › ijms-1229742-supplementary.pdf]
